# Between competence and warmth: the remaining place of the physician in the era of artificial intelligence

**DOI:** 10.1038/s41746-021-00457-w

**Published:** 2021-05-14

**Authors:** David Drummond

**Affiliations:** 1 Department of Pediatric Pulmonology and Allergology, University Hospital Necker-Enfants Malades, AP-HP, Paris, France; 2 INSERM UMR1138, Information Sciences to Support Personalized Medicine, Team Heka, Centre de Recherche des Cordeliers, University of Paris, Paris, France

**Keywords:** Medical ethics, Health policy

## Abstract

Competence and warmth are two essential dimensions of patient care. During the twentieth century, the industrial revolution in data collection, with the increasing use of machines and the division of labor that led to the development of many subspecialities, increased the overall competence of physicians at the expense of the warmth dimension. The spread of patient-centered care principles aimed to rebalance the two dimensions. In the twenty-first century, the industrial revolution in data processing with the emergence of algorithmic decision-making systems based on artificial intelligence is likely to disrupt further this balance. Competence will no longer be the prerogative of physicians, but a dimension to be shared between physicians and autonomous algorithmic decision-making systems, by contrast to warmth which should remain a human attribute. In this comment, we discuss the extent to which competence and warmth can remain the core dimensions of physician care in the era of artificial intelligence.

## Introduction

Patients need both competence, which refers to their physician’s ability to cure them using biomedical knowledge and scientific reasoning, and warmth, which refers to their physician’s ability to care for them by establishing an empathic relationship and considering them as unique individuals^1,2^. Competence has been described as cure-oriented, as opposed to warmth which is care-oriented, and can be associated with compassionate care^3^. Physicians are expected to provide both, with competence based on reason and objectivity, and warmth on emotion and subjectivity. Reconciling the two dimensions has always been a challenge. Even during the nineteenth century and the scientific revolution, the pure scientist Claude Bernard recognized that “the physician often finds himself obliged to take into account, in the treatments he prescribes, what is called the influence of morale on the physical body and consequently, a multitude of considerations of family or social position that have nothing to do with science”^4^. In this viewpoint, we discuss how the relative places of competence and warmth have been disrupted by the industrial revolution in data collection, and how they are likely to be further disrupted now by the industrial revolution in artificial intelligence (AI).

## The twentieth century and the industrial revolution in data collection

**Fig. 1:**
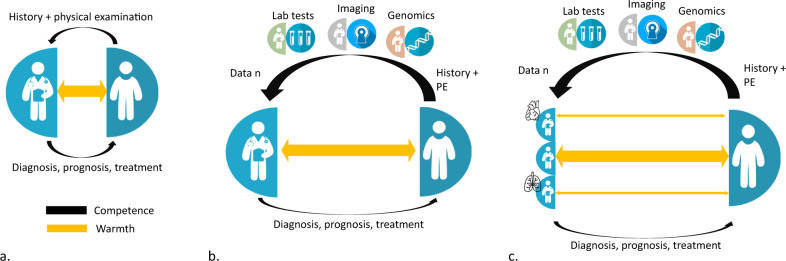
Competence and warmth in the era of data revolution. **a** Until the early twentieth century, physicians collected data from history and physical examination to diagnose conditions, estimate prognosis, and/or treat their patients (competence) while establishing a singular relationship with them (warmth). **b** The data revolution was marked by the increasing use of machines which led to a division of labor in data collection, with the involvement of biologists, radiologists, geneticists, etc. **c** The accumulation of knowledge led to the emergence of specialists and subspecialists, and medical decisions now often require multidisciplinary teams. Overall competence increased but the multiplication of organ specialists and the increasing place of machines threatened the physician–patient relationship. The concept of patient-centered care rebalanced competence and warmth. PE physical examination.

To establish a diagnosis, estimate a prognosis, identify the causes of diseases, and select treatments, i.e. the four fundamental tasks that can be gathered under the term of competence^1,5^, physicians must first gather information from their patients. Until the twentieth century, data collection was limited to the history and physical examination performed by physicians in the secrecy of their offices (Fig. 1a). Thereafter, data collection underwent its industrial revolution, with two features in common with previous industrial revolutions: the use of machines and the division of labor^6^.

The use of increasingly sophisticated machines has led to the industrial collection of objective and quantitative data, including physiological data from sensors (heart rate, temperature, oxygen saturation, blood pressure, etc.), biological data, imaging data, functional test data, and increasingly “omics” data (genomics, proteomics, etc.) (Fig. 1b). The best illustration of this proliferation of data sources is the management of patients in intensive care units, where patients are connected to multiple monitors, undergoing blood tests and imaging exams several times a day to achieve precision medicine. However, the industrial revolution in data collection is not limited to hospitals but extends to the homes of patients with chronic disease who are increasingly equipped with connected objects. For example, continuous glucose monitoring using a sensor worn on the skin is becoming the gold standard in diabetes management, and smartinhalers are increasingly used in the management of childhood asthma to record adherence to controller treatments and/or detect asthma attacks^7,8^.

The consequence of the multiplication of data sources is the division of labor, which represents the second characteristic of the industrial revolution in data collection. Whereas physicians used to collect all the necessary data themselves through history and physical examination, the data revolution led to the multiplication of stakeholders. Biologists, radiologists, geneticists, pathologists, and many other scientists are now involved in the collection and interpretation of data, while specialists (e.g. cardiologists) and subspecialists (e.g. interventional cardiologist, cardiac electrophysiologist, pediatric cardiac electrophysiologist) are required to gather all data and make complex decisions limited to a particular organ, a particular organ dysfunction, or a particular organ dysfunction in a particular population. The increasing level of complexity in medical decisions due to the proliferation of data sources and the need to involve many different specialists has shifted medical decisions from an individual process made by physicians in the secrecy of their offices to a collective process made in multidisciplinary meetings (Fig. 1c).

These data revolution increased the global competence of physicians, who could rely on more information to refine their diagnosis, prognosis, and better select the appropriate treatment^9^. For example, in cystic fibrosis patients, comprehensive data collection involving different specialists (geneticists, radiologists, organ specialists, etc.) made it possible to better distinguish between different classes of mutations (diagnosis), better anticipate when patients should be referred to a lung transplant center (prognosis), and propose personalized therapies (treatment), all leading to a dramatic improvement in patients’ lifespan^10^. However, if the competence of physicians increased, the revolution in data collection threatened their warmth. A first consequence was that if decisions could be made on the basis of remotely collected data, the doctor–patient relationship became optional, as Abraham Verghese^11^ stated: “iPatients are handily discussed in the bunker, while the real patients keep the beds warm and ensure that the folders bearing their names stay alive on the computer”. A second consequence was that the accumulation of specialists and subspecialists induced an organ-based vision of the patient, rather than a holistic view of the patient. At the end of the twentieth century, three French physicians underlined that “The pathology of the sick person is very quickly abandoned in favor of an organ pathology, itself quickly supplanted by the interest for a single dysfunction of this organ”^12^. Thus, several aspects of the physician “warmth” have been affected by the data revolution: active listening (“my doctor hears me”), because an increasing part of the information needed to make a diagnosis comes from complementary exams and not from patients’ explanations; passion for people (“my doctors practice to help people and loves what he/she does”), because of physicians losing the meaning of their practice and considering data collection repetitive and the standardization of patient management dehumanizing; and patient-specific warmth (“my doctor takes my perspective into account”), because of the predominant disease-centered approach that does not take into account the patients’ personal values and life context, which includes family members. In the face of this gradual dehumanization of medicine, the concept of “patient-centered care” emerged. Physicians were asked to personalize the care they delivered through better consideration of the person behind the disease and his/her family members, better communication, shared medical decisions, i.e. to provide more “warmth”^13^. As a result of this movement, many medical school curricula have been redesigned to help students provide warm, person-centered health care, and patient-centered care programs have been supported and increasingly implemented in hospitals and outpatient settings^14–16^. The relative positions of competence and warmth were in the process of being rebalanced when the AI revolution occurred.

## The twenty-first century and the industrial revolution in AI

After the first step of collecting data from their patients, the second step for physicians is to use their clinical reasoning to make medical decisions. Just as machines have become increasingly important in data collection in the last decades, they may become essential in medical decisions, limiting the role of physicians. Indeed, from an informatic point of view, clinical reasoning is data processing, data can be processed by algorithms, and algorithms are currently able to deliver a diagnostic probability, a prognostic estimation, or the selection of a treatment. Thus, AI can perform three of the four tasks usually attributed to physician competence^17^, with comparable or better performance than clinicians^18–20^.

If AI becomes as competent or more competent than physicians, the question becomes that of the physician’s remaining place. Indeed, the industrial revolution of AI follows the same path as the industrial revolution in data. Machines, here computers and their software, from electronic clinical decision support systems to autonomous algorithmic decision-making systems (AADMS), are taking an increasingly important place in medical decisions^21,22^. AAMDS may become new “actors” in the care of patients, making limited, pre-defined types of medical decisions. Indeed, there are repeated decisions without an emotional dimension, often already taken autonomously by the patients, such as the adjustments of the insulin dose in diabetes. These decisions can be successfully managed by AAMDS, as already demonstrated with artificial pancreas systems that track blood glucose levels using a continuous glucose monitor and automatically determine and deliver the appropriate dose of insulin when needed using an insulin pump. There is no longer any intervention of the physician in the choice of the dose to be administered, this competence having been transferred from humans to software^23^. By contrast, other decisions cannot be standardized and/or require warmth, such as the announcement of a serious illness, or any choice requiring an in-depth discussion of the patient’s values such as a transition from curative to palliative care. These decisions must remain the prerogative of physicians. Therefore, while the data revolution led to the emergence of multidisciplinary decisions, it is likely that the AI revolution will result in the definition of different levels of decisions, with some levels of decisions allowing AAMDS and others not.

Again, the second characteristic of the AI revolution is the division of labor: in addition to the support staff already existing in health care (technicians, statisticians, etc.), different specialists in data science and informatics are now required to implement additional databases, ensuring data quality, developing models based on machine learning techniques, integrating these models in electronic clinical decision support systems or AAMDS, and creating the appropriate interface with end-users. Unlike in the early twentieth century, when physicians provided 100% of patient care, their place in the early twenty-first century is likely to become increasingly limited with the intervention of dozens of actors responsible for data collection (biologists, radiologists, geneticists, pathologists, etc.) and data processing (data scientists, informaticians, etc.). Beside these humans, AAMDS and other software may be considered as new “actors” in this new area of medicine^24^. This vision is in line with the “fourth industrial revolution” which corresponds to the trend towards automation in manufacturing technologies, with the production of smart machines that can analyze and diagnose issues without the need for human intervention^25^.

**Fig. 2:**
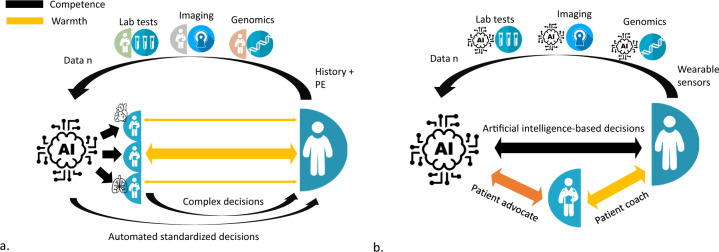
Competence and warmth in the era of artificial intelligence. **a** In the first hypothetical scenario, physicians remain the conductors: they are the ones who make complex decisions involving patient values, and who prescribe the most appropriate autonomous algorithmic decision-making systems among the different available ones to delegate decisions that can be standardized or without emotional dimension. **b** In the second hypothetical scenario, physicians are overwhelmed by the performance of artificial intelligence. They are hired by digital giants to tailor the algorithms to patients’ values, thus becoming their advocate, and accompany the decisions made by the artificial intelligence system, coaching their patient towards better health. PE physical examination.

The future will tell us how physicians can deal with this situation on the long term. Two scenarios can be considered. In the first scenario, physicians remain the conductors (Fig. 2a). They continue to prescribe tests, to seek expert advices when necessary, and become AADMS prescribers, choosing with their patients the most appropriate AADMS based on their diseases, characteristics, and values. From the physicians’ perspective, this scenario would be equivalent to that prevailing in evidence-based medicine^26^: based on the evidence found in the literature comparing the performance of different AADMS, their experience with previously prescribed AADMS, and patient preferences and values, physicians would choose the best AADMS for each patient. In this scenario, physicians would continue to combine competence, which becomes their ability to select the appropriate AADMS, and warmth with patient-centered care and an empathetic relationship. The only difference with the current situation is that physicians will no longer prescribe specific treatments but rather AADMS, i.e. systems that themselves determine the most appropriate combinations and/or sequences of treatments and update their decisions after evaluating the results of their previous decisions.

In the second scenario, physicians are overwhelmed by the high performance of AADMS developed by the digital giants and become hired by these companies to be at the interface between AADMS decisions and patients (Fig. 2b). With AADMS, or their human representatives the “health engineers”, physicians would become their patients’ advocates, transmitting their vision of life and values so that AADMS decisions can be tailored to their unique patient. With patients, physicians would announce important diagnoses made by AADMS and accompany patients towards better health as a coach would. Several companies could soon offer their digital ecosystem to patients, including the products and machines needed for data collection, the teams required for data processing, and the accompanying physicians, with the risk of a new organization corrupted towards the profits of these companies. Only large companies are currently able to cover the entire health chain, which a century ago was the monopoly of physicians. In this scenario, competence, defined as the ability to establish a diagnosis, estimate a prognosis, or select a treatment would no longer be the prerogative of the physician. The role of physicians would remain limited to the “warmth” dimension because they would accompany a care they would not master. However, even this “warmth dimension” is threatened by the progress made in the new domain of artificial emotional intelligence and its three main areas of research, i.e. recognition, generation, and augmentation of emotions^27^. Building on this research, empathetic conversational bots have been designed to establish long-term, social-emotional relationships with their users^28^. And indeed, humans are increasingly “trusting” these virtual agents, considering them over time as friends or companions^29,30^. Even in settings where patients require the most humanistic care such as in palliative care, empathetic conversational bots, capable of assessing and providing advice to reduce social isolation and unmet spiritual needs according to their creators, are being evaluated^31^.

## Conclusion

The industrial revolution in data collection and then data processing/AI has led to the multiplication of actors involved in patient care, these actors being no longer limited to humans but also to software that makes medical decisions. Physicians must keep the control over these new technologies if they want to keep the “competence” dimension on their side. Otherwise, they risk being hired by companies for their “warmth”, i.e. their ability to support patients in the digital ecosystem to which they have subscribed, at least for the time when artificial emotional intelligence is still in its infancy. This would sign the loss of medical competence as currently defined, and the return of physicians in the pre-scientific revolution era when they practiced a discipline they did not master.

## References

[1] Howe LC, Leibowitz KA, Crum AJ (2019). When your doctor ‘gets it’ and ‘gets you’: the critical role of competence and warmth in the patient-provider interaction. Front. Psychiatry.

[2] Good, B. J. & Good, M.-J. D. Learning medicine: the constructing of medical knowledge at Harvard Medical School. *Knowl. Power Pract. Anthropol. Med. Everyday Life* 81–107 (1993). https://rl.talis.com/3/ucl/items/156C1EE9-87C4-D087-BC4A-24307CE31315.html.

[3] De Valck C, Bensing J, Bruynooghe R, Batenburg V (2001). Cure-oriented versus care-oriented attitudes in medicine. Patient Educ. Couns..

[4] Entralgo, L. *Le medecin et la malade* (Hachette, 1969).

[5] Cassel EJ (1998). The nature of suffering and the goals of medicine. Loss Grief Care.

[6] Durkheim, E. *The Division of Labor in Society* (Simon and Schuster, 2014).

[7] Klonoff DC (2005). Continuous glucose monitoring: roadmap for 21st century diabetes therapy. Diabetes Care.

[8] Ramsey RR, Guilbert TW (2021). Exciting era of sensor-based electronic monitoring of adherence in pediatric asthma. Pediatrics.

[9] Young LB (2011). Impact of telemedicine intensive care unit coverage on patient outcomes: a systematic review and meta-analysis. Arch. Intern. Med..

[10] Bell SC (2019). The future of cystic fibrosis care: a global perspective. Lancet Respir. Med..

[11] Verghese A (2008). Culture shock—patient as icon, icon as patient. N. Engl. J. Med..

[12] Mattei, J.-F., Étienne, J.-C. & Chabot, J.-M. *De la médecine à la santé: pour une réforme des études médicales et la création d’universités de santé* (Flammarion, 1997).

[13] Laine C, Davidoff F (1996). Patient-centered medicine: a professional evolution. JAMA.

[14] Audet A-M, Davis K, Schoenbaum SC (2006). Adoption of patient-centered care practices by physicians: results from a national survey. Arch. Intern. Med..

[15] Härter M (2017). The long way of implementing patient-centered care and shared decision making in Germany. Z. F.ür. Evidenz Fortbild. Qual. Im. Gesundheitswesen.

[16] Kuo DZ (2012). Family-centered care: current applications and future directions in pediatric health care. Matern. Child Health J..

[17] Hatherley JJ (2020). Limits of trust in medical AI. J. Med. Ethics.

[18] Shen J (2019). Artificial intelligence versus clinicians in disease diagnosis: systematic review. JMIR Med. Inform..

[19] van Doorn WPTM (2021). A comparison of machine learning models versus clinical evaluation for mortality prediction in patients with sepsis. PLoS ONE.

[20] Tyler NS (2020). An artificial intelligence decision support system for the management of type 1 diabetes. Nat. Metab..

[21] Bright TJ (2012). Effect of clinical decision-support systems. Ann. Intern. Med..

[22] Goodman B, Flaxman S (2017). European Union Regulations on algorithmic decision-making and a “right to explanation”. AI Mag..

[23] Doyle FJ, Huyett LM, Lee JB, Zisser HC, Dassau E (2014). Closed-loop artificial pancreas systems: engineering the algorithms. Diabetes Care.

[24] Levy, F. & Murnane, R. J. *The New Division of Labor: How Computers Are Creating the Next Job Market* (Princeton University Press, 2012).

[25] The Fourth Industrial Revolution: what it means and how to respond. *World Economic Forum.* https://www.weforum.org/agenda/2016/01/the-fourth-industrial-revolution-what-it-means-and-how-to-respond/.

[26] Sackett DL, Rosenberg WMC, Gray JAM, Haynes RB, Richardson WS (1996). Evidence based medicine: what it is and what it isn’t. BMJ.

[27] Schuller D, Schuller BW (2018). The age of artificial emotional intelligence. Computer.

[28] Bickmore TW, Picard RW (2005). Establishing and maintaining long-term human-computer relationships. ACM Trans. Comput. Hum. Interact..

[29] Zhou L, Gao J, Li D, Shum H-Y (2020). The design and implementation of XiaoIce, an empathetic social Chatbot. Comput. Linguist..

[30] Fan, L. et al. Do we need emotionally intelligent artificial agents? *First Results of Human Perceptions of Emotional Intelligence in Humans Compared to Robots* 129–141 (Springer International Publishing, 2017). 10.1007/978-3-319-67401-8_15.

[31] Boston Medical Center. *Conversational Agents to Improve Quality of Life in Palliative Care*. https://clinicaltrials.gov/ct2/show/NCT02750865 (2021).

